# Localization of Physical Activity in Primary School Children Using Accelerometry and Global Positioning System

**DOI:** 10.1371/journal.pone.0142223

**Published:** 2015-11-04

**Authors:** Rahel Bürgi, Laura Tomatis, Kurt Murer, Eling D. de Bruin

**Affiliations:** Department of Health Sciences and Technology, Institute of Human Movement Sciences and Sport, ETH Zurich, Zurich, Switzerland; University of Leeds, UNITED KINGDOM

## Abstract

**Background:**

Ecological approaches have highlighted the importance of the built environment as a factor affecting physical activity. However, knowledge on children’s activity patterns is still incomplete. Particularly, data on the spatial context of physical activity is limited, which limits the potential to design location-based interventions effectively. Using global positioning system (GPS) and accelerometry, this study aimed to identify locations where children engage in moderate-vigorous physical activity (MVPA).

**Methods:**

Participants included 119 children (11–14 years, 57% girls) from public schools in Winterthur, Switzerland. During a regular school week between February and April 2013, children wore an accelerometer and GPS sensor for seven consecutive days. Time-matched accelerometer and GPS data was mapped with a geographic information system and each data point was assigned to one of seven defined activity settings. Both the absolute amount of MVPA and proportion of time in MVPA were calculated for every setting. Multilevel analyses accounting for the hierarchical structure of the data were conducted to investigate any gender differences.

**Results:**

Children achieved most MVPA on streets (34.5%) and on school grounds (33.4%). The proportion children spent in MVPA was highest in recreational facilities (19.4%), at other schools (19.2%) and on streets (18.6%). Boys accumulated significantly more MVPA overall and on other school grounds (*p* < 0.05) and showed a significantly higher proportion of time in MVPA at own school and outside of Winterthur (*p* < 0.05).

**Conclusions:**

The results indicate the importance of streets and school grounds as activity-promoting environments. The high use of streets may be an indicator for active transportation, which appears to contribute to an active lifestyle in both genders. In contrast, the school setting is more likely to encourage physical activity in boys. Recreational facilities seem to be conducive for MVPA among both genders, although infrequently visited during the week of measurement.

## Introduction

Physical activity (PA) is associated with many health benefits and widely recognized as an important factor for children’s physical, psychological and social development [1–3]. When performed during childhood, PA may also contribute to an active lifestyle in adulthood [4, 5] and, thus, provides long-term protective effects on health [5]. Despite these benefits, global trends indicate alarmingly low levels of PA among children when compared to recommended levels [6–8]. Studies reported that very few children across Europe met the recommendations of 60 minutes in moderate-vigorous physical activity (MVPA) per day [6, 8]. In Switzerland, only 20% of the boys and 11% of the girls aged 11 years reported at least one hour of MVPA daily and were categorised as adequately physically active according to the PA recommendations [8].

In accordance with this high inactivity, the worldwide prevalence of childhood overweight and obesity is still increasing or has stabilized at a high level [9, 10]. About one in five children in Switzerland is overweight or obese [11]. Therefore, overweight and obesity in youth remains a major public health concern and further policies that promote PA among children are urgently needed.

In recent years, ecological approaches towards PA promotion have generated much interest among researchers and in public health [12]. Besides individual and interpersonal factors, the physical environment has been recognized as an important determinant of PA and has become an increasing focus of research [13, 14]. Although findings are currently inconsistent [13, 15], a growing number of studies emphasise that built environment factors such as walkability, traffic speed and volume, access to recreation facilities, land-use mix and residential density are generally associated with levels of PA [16]. Therefore, recent changes in the built environment due to urbanisation could play a crucial role in the low level of PA [17].

Challenges in the measurement of PA and difficulties of quantifying actual locations where PA takes place are two main reasons for these inconclusive results [15, 18]. Many studies on this topic have used self-reporting techniques such as questionnaires and activity diaries, or direct observation to assess the association between PA and built environment. However, inference is limited with these methods since self-reporting is subjective and may suffer from recall bias, whereas the assessment of an individual’s total PA might not always be possible by direct observation. Limited inference is also present in studies using GIS, in which ecological fallacy can be a major issue. Ecological fallacy can occur when deriving conclusion about individuals solely based on the aggregate statistics of an area [19].

The development of lightweight, accurate and affordable Global Positioning System (GPS) devices has opened up new opportunities to gain more knowledge about the influence of neighbourhood structure on PA. By continuously recording location, GPS allows objective measurement of how people move within their neighbourhood [20, 21]. In recent years, some researchers have begun to combine GPS receivers with accelerometers, allowing assessment of PA and the spatial context in which it is carried out [19]. Studies showed that GPS devices combined with accelerometers are able to track a participant’s movement pattern accurately [22, 23]. Therefore, the combination of accelerometry and GPS is a promising method to gain further insights into the spatial context of PA.

Little is known about the setting in which children are physically active [15, 18, 19], although this information would be of great importance for future location-based programs and policies to promote PA. To date, no study based on objective measures and describing the spatial activity patterns of children in Switzerland has been published. The purpose of this study was to use the novel combination of GPS and accelerometry to identify locations where children spend time and engage in PA. In particular, we aimed to determine both the amount of MVPA and the proportion of time spent in MVPA in different activity settings and across genders.

## Methods

### Participants and setting

The study was conducted between February and April 2013 in the municipality of Winterthur, which is the sixth largest city in Switzerland with a population of over 100,000 residents. Winterthur is located in the German-speaking part of Switzerland and represents an urban area which is representative in relation to demographic values such as unemployment rate, foreign nationals or social assistant rate [24]. All teachers from a public school in Winterthur in charge of a sixth grade class (n = 47) were invited to participate in the present study. If they agreed to participate, classes were visited and briefed about the study. Participation was voluntary and children were provided with an information letter for their parents. Parental written informed consent was obtained along with child consent. The ability to engage in usual everyday PA and the absence of any severe disabilities were the only inclusion criteria. The study was approved by the ethics committee of ETH Zurich (EK 2012-N-62).

### Instruments and measures

PA was measured every ten seconds with a tri-axial accelerometer (GT3X, Actigraph, Pensacola, FL, USA). Given that cut-off points for different activity levels are based on the vertical axis, only data from this axis was included in the analysis. A short sampling interval was used to accurately capture children’s PA pattern, which is characterized by short, intermittent bursts of activity [25]. Simultaneously, a GPS receiver (BT-Q1000XT, QStarz, Taipei, Taiwan) recorded the geographical location at ten second intervals whenever there was sufficient satellite signal (Circular Error Probability CEP (50%) < 3m). A recent study by Schipperijn and colleagues [26] showed that this device has an acceptable dynamic spatial accuracy for use in larger population studies with a data collection period of seven or more days. In their experiment, 79% of data fell within 10m of the expected location.

All participating classes were visited during a regular school hour one day before the start of the measurement to distribute the devices among children and to provide them with updated information. Each child received an elastic belt equipped with an accelerometer and a GPS sensor. Participants were asked to wear the belt around the waist from waking time to bedtime on seven consecutive days starting the next day. Each child was given a charger for the GPS device and was instructed to charge the device during night when asleep. Children were asked to complete a small diary throughout the week reporting times they woke up and went to bed and times and reasons when monitors were not worn. In addition, age, gender, nationality and home address were assessed and height and weight were measured using a measurement tape and a digital scale (Beurer GS 12, Beurer GmbH, Ulm, Germany) to the nearest 0.5cm and 0.1kg, respectively. Nationality was defined as the number of parents with a Swiss nationality and subdivided into three categories (e.g. none, one or two of the parents with Swiss nationality).

### Data merging and processing

Each participant’s GPS and accelerometer data was manually reviewed to ensure that both files contained adequate data. The GPS and accelerometer files were then matched by time using existing software (Actilife 6.5.2, Actigraph, Pensacola, FL, USA) producing a measure of activity and location for each recorded GPS point.

The processing of the matched data was performed using MATLAB R2012a (MathWorks, Massachusetts, USA) and R v2.15.2 (R Development Core Team, Vienna, Austria). Intervals with > 60 minutes of consecutive zero activity counts were classified as non-wear time and excluded from analysis [27]. Activity records > 5461 counts per ten seconds were identified as outliers [28] and replaced with the mean of the previous and the following value. Each data point was then classified into one of the four intensity categories sedentary (< 101 counts per minute (CPM)), light (101–2295 CPM), moderate (2296–4011 CPM) or vigorous (≥ 4012 CPM), based on age-appropriate cut-off points [29, 30]. Furthermore, the data was processed by visual observation as well as automatic identification of invalid GPS data points using extreme changes in distance and invalid values of altitude and removing them from data.

### Activity settings

**Fig 1:**
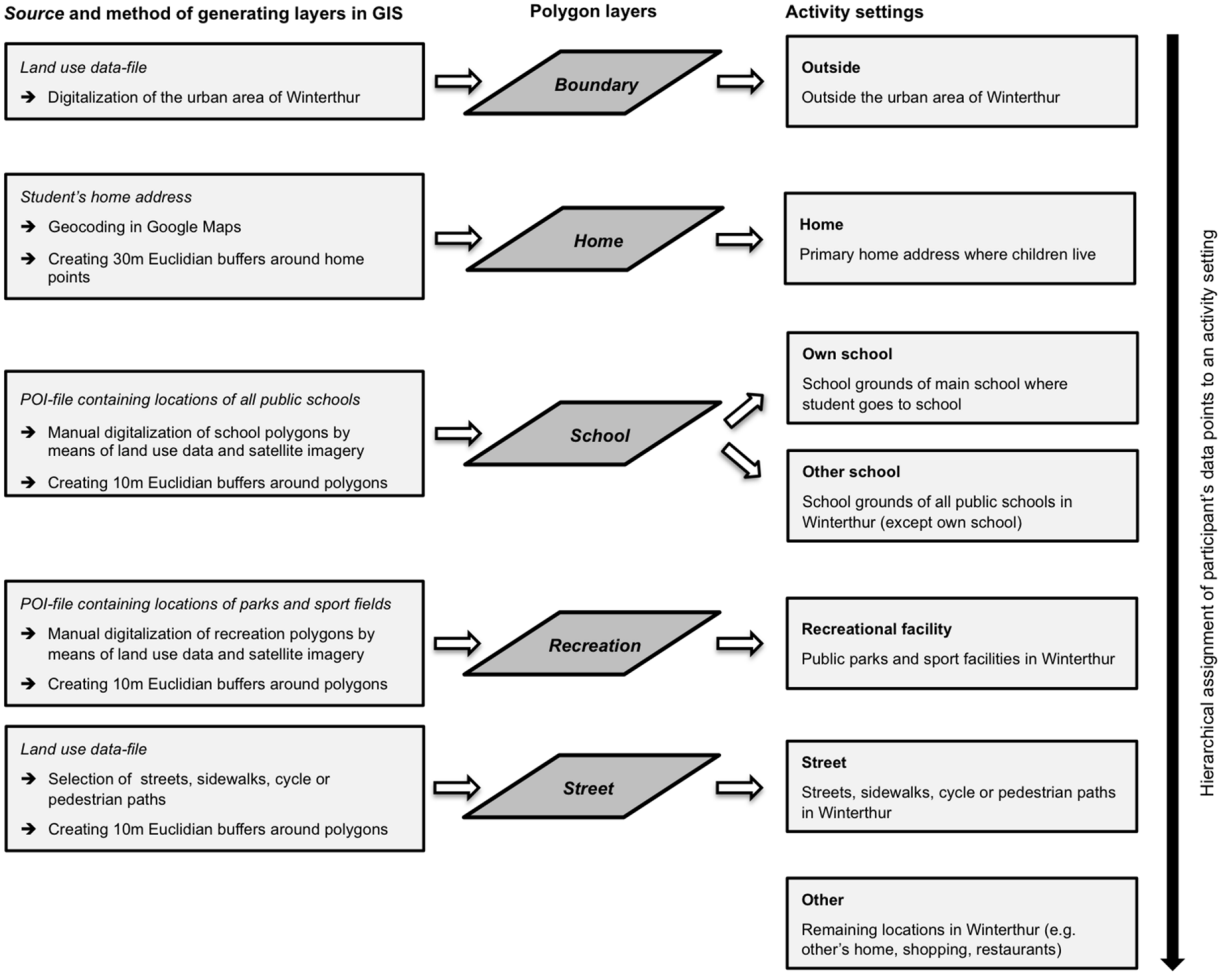
Definitions and methods of generating the seven activity settings. Definitions of the seven activity settings and overview of their construction in a geographic information system (GIS). Land use data and points-of-interest (POI) were provided by the Land Surveying Office of Winterthur. The assignment of participant’s data points to one of the seven activity settings was carried out in a hierarchical top-down order.

The setting-based categorisation of the matched data points was conducted in ArcGIS 10 (ESRI, Redlands, CA, USA) using further geospatial data (land use data, street network and points-of-interest-file), which was provided by the Land Surveying Office of Winterthur. Based on prior studies [15, 19, 31] and the ability to clearly assign each recorded GPS point to a precise location by using ArcGIS, we chose to define seven activity settings: home, own school, other school, recreational facility, street, other, and outside. The definitions and methods of generating the seven settings are presented in Fig 1.

Each participant’s data points were imported into ArcGIS and plotted on a separate layer. To assign a data point to one of the seven activity settings, we used the point-in-polygon overlay in which each participant’s point layer was overlaid on five different polygon layers defined in Fig 1. This overlay was carried out in a hierarchical order by firstly assigning points that fell outside Winterthur, followed by points that fell within the home buffer, school polygons, recreational facility polygons and street polygons. Lastly, all remaining data points were categorized as other. For school, recreational facility and street polygons, we chose a buffer zone of ten meters to take the measurement error of the GPS devices into account [23, 26, 32].

### Data Analysis

Only matched wear-time data was used for analysis. To best capture all PA settings where children spent time and because no clear standard criteria for the amount of measurement time per observation day exists, we determined an average value from a criterion of one day with at least one hour [19] and at least three days with a minimum of four hours [28]. Thus, the children had to provide at least one day with four full hours of matched data to be included in the analysis.

As outcome measures, total time per week (in hours) and MVPA per week (in minutes) were calculated for each setting and participant. Moreover, the proportion of time spent in MVPA (in %) out of the total time spent in each setting was calculated.

Statistical analyses were performed in R. Descriptive statistics were used to calculate frequency distributions (number (n) and proportion (%)), mean and standard deviation (SD), or median and interquartile range (IQR) to describe the general characteristics of the study population. As the outcome measures were not normally distributed, median and IQR were used to present total time, the amount of MVPA and proportion of time spent in MVPA. We used multilevel analyses accounting for the hierarchical structure of the data in which individuals and schools were included as random effects [33]. Models were transformed by log transformation to fulfil the model assumptions. To adjust for individual differences, wear time, nationality and week day were included as potential confounders, as these parameters may have an influence on children’s activity patterns [34, 35]. A backward elimination algorithm with Akaike’s information criterion (AIC) as goodness-of-fit measure was applied to test the contribution of the entered predictors. To explore interactions between setting and gender, we finally used a single-step-method to calculate contrasts between boys and girls in every setting. Gender differences were provided by the transformed and adjusted *p*-values.

## Results

A total of 123 out of 213 invited children provided consent and wore a measurement belt. Two participants had to be excluded due to abnormal accelerometer data and another two did not fulfil the inclusion criteria of at least one valid day with four hours of combined data. Thus, the final study population consisted of 119 children from ten different classes, including 51 boys and 68 girls. Table 1 presents the general characteristics of the study population. In total, participants wore the accelerometer and GPS device during a daily median of 12.7 hours at an average of 6.5 days (SD: 1.2). Out of seven possible days, participants reached a mean of 5.8 valid days (SD: 1.7) during the measurement week. After removing a total of 65 782 invalid GPS points during the data cleaning (2.6% of the total matched wear-time data), the GPS location was available for a median of 74.7% (IQR: 60.0–85.7) of the total accelerometer wear-time data.

**Table 1:** General characteristics of the study population.

	Total	Boys	Girls
**Population (%)**	119 (100.0)	51 (42.9)	68 (57.1)
**Mean age in years (SD)**	12.5 (0.4)	12.5 (0.5)	12.5 (0.3)
**Mean body height in cm (SD)**	154.9 (7.6)	153.7 (7.7)	155.9 (7.5)
**Mean body weight in kg (SD)**	45.6 (9.2)	45.4 (10.3)	45.8 (8.3)
**Mean BMI in in kg/m** ^**-2**^ **(SD)**	18.9 (3.1)	19.1 (3.4)	18.8 (2.9)
**Median daily wear time in h (IQR)**	12.7 (11.5–13.5)	12.8 (11.1–13.5)	12.7 (11.5–13.4)
**Median daily hours of combined data (IQR)**	9.3 (6.7–10.6)	9.4 (7.9–10.7)	9.3 (6.6–10.5)

SD, Standard Deviation; BMI, Body Mass Index; IQR, Interquartile Range

### Total time

During the measurement week, all children spent time at home and at own school, on streets and in other places. Four participants (3.4%) did not visit recreational facilities, eight participants (6.7%) did not record any time at other schools and 75 children (63%) did not leave the City of Winterthur.

**Fig 2:**
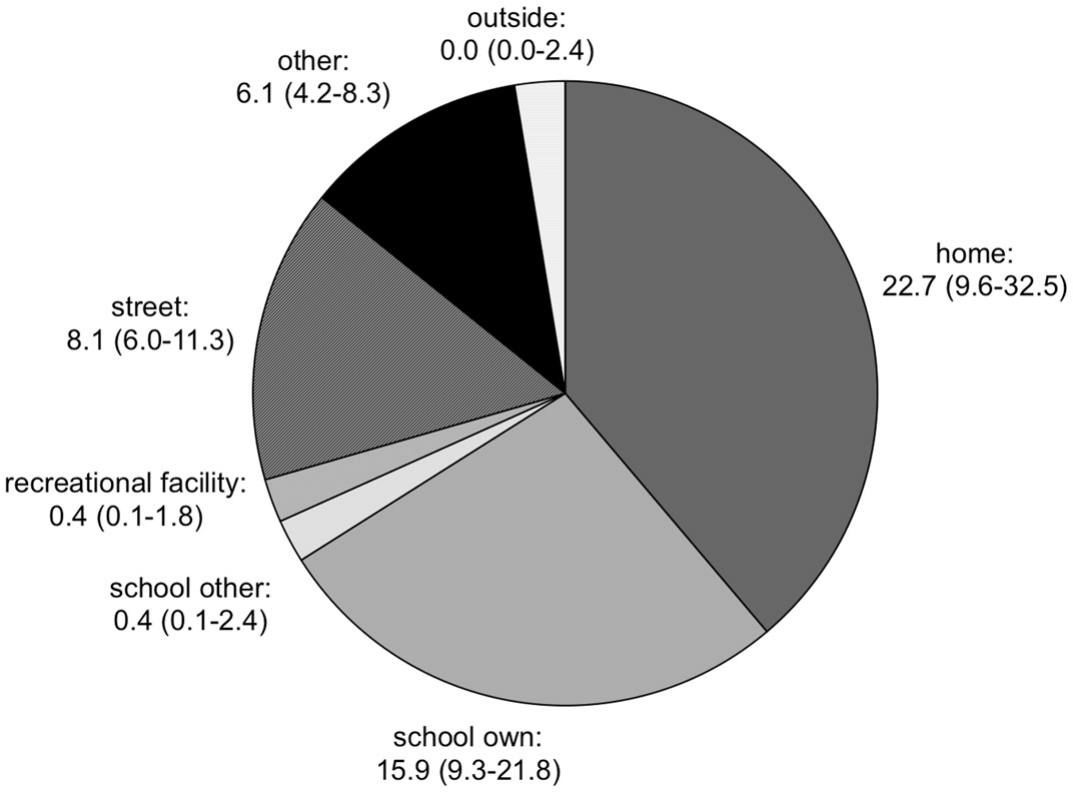
Total weekly time spent per setting. Total time (in hours) spent in each activity setting during the measurement week. Since boys and girls did not show any significant differences in total time spent in a setting, data is presented for the whole study sample using median and interquartile range (IQR).

Children spent 38.8% of their time at home (22.7 hours; IQR: 9.6–32.5), 27.2% at their own school (15.9 hours; IQR: 9.3–21.8) and 15.3% on streets (8.1 hours; IQR: 6.0–11.3). Only 2.3% were recorded in recreational facilities (0.4 hours; IQR: 0.1–1.8) and at other schools (0.4 hours; IQR: 0.1–2.4). The remaining 11.6% (6.1 hours; IQR: 4.2–8.3) were spent in other places such as other’s homes, shopping malls or woodland (Fig 2). Gender did not have a significant influence, neither on total time recorded nor on time spent in any setting (all *p* > 0.05).

### MVPA time

Table 2 provides the results on the amount of MVPA accumulated overall and in the seven activity settings for the total population and separated by gender. Over all settings, children accumulated 296.7 minutes of MVPA during the measurement week. Boys were significantly more active than girls were during the week, accumulating 63.9 more minutes in MVPA than girls (*p* < 0.001). The most MVPA was accumulated on streets (34.5%) and at own school (26.8%). Street was the only setting in which girls accumulated slightly, albeit not significantly, more MVPA than boys (*p* > 0.999). In all remaining settings, boys achieved a higher amount of MVPA than their female peers did. However, we only found a significant gender difference at other schools with boys reporting 11.2 more minutes in MVPA (*p* = 0.035) and a non-significant trend at own school with girls spending 8.4 minutes less in MVPA (*p* = 0.053).

**Table 2:** Weekly minutes of MVPA by gender.

	Total (N = 119)		Boys (n = 51)		Girls (n = 68)		
	Mdn	IQR	Mdn	IQR	Mdn	IQR	*p*-value
**Total**	296.7	(221.8–384.5)	348.5	(255.3–441.7)	284.6	(202.3–317.6)	**<0.001**
**Home**	34.0	(18.5–59.0)	41.5	(20.2–60.2)	30.8	(17.3–55.3)	0.476
**Own school**	74.7	(51.2–108.3)	80.3	(58.2–136.7)	71.9	(40.1–91.8)	0.053
**Other school**	3.7	(0.3–29.0)	14.5	(0.2–45.8)	3.3	(0.8–24.3)	**0.035**
**Recreational facility**	4.7	(0.3–19.8)	9.2	(1.2–24.7)	2.7	(0.3–13.3)	0.430
**Street**	94.3	(57.0–143.7)	91.0	(54.8–145.0)	99.0	(65.2–139.5)	>0.999
**Other**	25.2	(14.3–39.2)	25.5	(12.5–48.7)	24.7	(16.2–36.1)	>0.999
**Outside**	0.0	(0.0–4.2)	0.0	(0.0–15.7)	0.0	(0.0–1.8)	0.391

Bold: significant difference *p* < 0.05.

IQR, Interquartile Range; Mdn, Median; MVPA, Moderate-Vigorous Physical Activity

### Proportion of time spent in MVPA

Overall, the children recorded 8.4% of the time in MVPA, with boys spending only a slightly higher proportion of time in MVPA compared to girls (9.7% vs.7.2%; *p* = 0.189). The proportion of time spent in MVPA was largest in recreational facilities (19.4%), at other schools (19.2%) and on streets (18.6%). A considerably smaller proportion was classified as MVPA when visiting own school (8.6%), other places (7.1%) and places outside of Winterthur (5.2%). The proportion of time in MVPA at home (3.0%) was even smaller (Table 3). For six out of the seven activity settings, the proportion of time in MVPA was higher among boys than among girls, whereby significant differences were found at own school (3.4%, *p* = 0.049) and out of study area (6.9%, *p* = 0.009). Moreover, a non-significant trend was found at other schools with boys being more active than girls were (8.8%, *p* = 0.072). Street was the only setting where girls showed a slightly higher proportion of time in MVPA than boys (19.1% vs. 17.9%), however, this difference was not statistically significant (*p* = 0.477).

**Table 3:** Proportion (%) of time spent in MVPA by gender.

	Total			Boys			Girls			
	N	Mdn	IQR	n	Mdn	IQR	n	Mdn	IQR	*p*-value
**Total**	119	8.4	(6.5–11.2)	51	9.7	(7.7–12.6)	68	7.2	(6.2–9.9)	0.189
**Home**	119	3.0	(1.9–4.2)	51	3.3	(2.1–4.6)	68	2.8	(1.8–3.9)	>0.999
**Own school**	119	8.6	(5.8–11.7)	51	10.5	(8.3–13.3)	68	7.1	(5.1–9.7)	**0.049**
**Other school**	111	19.2	(8.5–33.2)	46	26.1	(12.5–33.5)	65	17.3	(8.2–29.0)	0.072
**Recreational facility**	115	19.4	(6.1–33.6)	48	19.5	(6.2–32.8)	67	18.2	(5.0–36.2)	>0.999
**Street**	119	18.6	(12.3–26.9)	51	17.9	(10.8–26.6)	68	19.1	(13.9–27.5)	0.477
**Other**	119	7.1	(4.9–10.1)	51	7.6	(5.1–11.2)	68	6.8	(4.8–8.5)	0.955
**Outside**	44	5.2	(2.3–12.7)	22	10.5	(4.0–15.3)	22	3.6	(2.0–5.4)	**0.009**

Bold: significant difference *p* < 0.05.

IQR, Interquartile Range; Mdn, Median; MVPA, Moderate-Vigorous Physical Activity

## Discussion

The purpose of this study was to use the novel combination of GPS and accelerometry to identify locations where children spend time and engage in PA. In particular, we aimed to determine both the amount of MVPA and the proportion of time spent in MVPA in different activity settings and across genders. To the best of our knowledge, there is no study published that assessed children’s spatial activity pattern by means of accelerometer and GPS device in Switzerland.

The main finding is that children achieved most of their MVPA on streets and school grounds. This indicates the importance of these settings as places where children are able to be physically active. The finding is in line with a recent review reporting that time spent on streets contributed the largest portion of MVPA, followed by school grounds and home setting [36]. When taking into account the total time spent in a setting, we found, in support of previous research, that the proportion of time spend in MVPA was highest in recreational facilities [31, 37, 38]. The significant gender difference on school grounds, with boys being more active than girls, confirms previous findings that the school setting is more likely to encourage boys to be physically active [32, 39, 40].

Combining both school settings, children recorded 30% of their time on school grounds and achieved 33% of their MVPA at school. This result demonstrates the importance of the school setting with regard to the daily accumulation of MVPA [15, 31, 32, 34]. Due to a high amount of regular class time spent sitting inside the school buildings [32, 40], the proportion of time spent in MVPA at own school was only slightly larger than the overall proportion of time spent in MVPA. However, the high proportion recorded at other schools supports recent research that stressed the importance of the school setting as an activity-promoting environment during recess and non-school hours [32, 35, 40]. The high proportion spent in MVPA at other schools can also be explained by the fact that Swiss sports clubs often use the school infrastructure for their training.

In line with the present study, previous research found that boys were more physically active in the school setting than girls [32, 39, 40]. This finding supports the assumption that girls either do not perceive this setting as equally enjoyable as boys do [39] or perceive different barriers that prevent them from being physically active [41]. Blatchford et al. [42] further reported that boys showed different preferences in schoolyard activities compared to girls. Boys were more likely to be engaged in ball games and vigorous play, whereas girls were more likely to be involved in skipping games, sedentary play, or social conversation. The different stage of maturation, with girls being less active due to the earlier maturation, could be another explanation for the observed gender difference [32, 43]. In this regard, future interventions to promote PA have to consider organizing school grounds in a way that also girls will be encouraged to be physically active.

Children spent around 15% of their recorded time on streets and accumulated 35% of their MVPA in this setting, which is in line with previous research [15, 18, 19]. This high use may be an indicator for active transportation and underlines the importance of active commuting to reach the daily recommended level of PA [31, 44, 45]. A recent study by Bringolf-Isler and colleagues [46] found a high proportion of active travellers (78%) and concluded that active commuting still is very prominent in Switzerland. Accordingly, almost all of our participants reported to usually walk or cycle to school. Rainham and colleagues [15] found that suburban girls achieved more time in MVPA while commuting than suburban boys. The present study supports this finding by the observation that streets were the only setting in which girls accumulated slightly more minutes in MVPA than boys. Children might also use streets for undertaking informal PA that contributes to an active lifestyle such as playing tag, skipping or ball games [18]. Given that streets are an important activity space used by both girls and boys aged 11 to 13, strategies to maintain or increase active commuting and to provide safe residential streets may be an important approach to promote PA in both genders [18].

Recreational facilities such as public parks, playgrounds or sport facilities were rarely visited during the measurement week. Previous studies already reported that the use of green spaces and playgrounds is low [31, 36]. In particular, recent research showed that parks and playgrounds are more likely to be visited in summer than in winter and spring, which is when the present study was conducted [19, 38]. Despite the limited use, the proportion of time spent in MVPA when visiting a park or sport facility was relatively high (19.4%). Klinker and colleagues [31] found comparable proportions for sport facilities (17.9%), playgrounds (28.2%) and urban green spaces (17.1%). A momentary epoch-level analysis in children aged 8–14 further demonstrated that a temporary exposure to greenness was associated with higher odds of being physically active [28]. The observed high proportion of time spent in MVPA confirms the assumption that such environments are highly conducive for PA among children even though rarely visited. Strategies to increase the use of parks and sport facilities may therefore be a promising option to promote PA among children. Additionally, the finding demonstrates the particular significance of distinguishing between total amount of MVPA and proportion of time spent in MVPA as outcome measure in further studies.

### Limitations

Although the combination of accelerometry and GPS seems to offer sensitive and accurate measures, there are several limitations. Firstly, known problems with waist-worn accelerometers are associated with the underestimation of activities in which the body centre of gravity is relatively fixed such as cycling or movements of the upper body [47]. Twenty-nine children engaged in sports could not wear the devices during their training or competition. Although only six children reported such an event more than once a week, this loss of recorded PA possibly has resulted in incomplete measures and underestimations, which predominantly affected recreational facilities and the school setting. The choice of processing methods and threshold values to determine MVPA may also have an impact on the recorded PA level [48]. Reactivity to measurement devices could lead to an unnaturally high level of PA. Although our participants did not show an especially high activity level on the first measurement day as reported in previous studies [49], this could have affected their behaviour during the whole measurement week. Furthermore, certain environments such as urban canyons, vegetative cover or indoors within structures impermeable to satellite signals can lead to inaccurate or missing GPS positions [15]. By identifying invalid GPS points and choosing a buffer zone of ten meters around polygons, we tried to reduce issues with spatial inaccuracy, but under- or overrepresentation of certain activity settings due to missing or misclassified GPS points cannot be completely excluded. In particular, the use of buffer zones accounting for the measurement error of the GPS devices might have generated new misclassifications, especially affecting the street setting. Consequently, the development of clear standards, which ensure more consistency in data collection and processing procedures between studies based on accelerometry and GPS is necessary. The current study was conducted during late winter and early spring with rather cold and rainy weather conditions. Bringolf-Isler and colleagues [44] showed that the time spent in different activities such as vigorously playing outdoors or biking significantly decreases with the sum of precipitation, whereas sedentary time spent indoors increases. Bad weather conditions may also be a reason not to participate in outdoor activities during recess [41]. Several studies further demonstrated that children use different activity settings to achieve PA during different seasons [19, 38]. Accordingly, our findings are not representative for the entire year. The study sample derived from one city in the German-speaking part of Switzerland. The included schools were public schools situated in different districts and distributed throughout the whole city. Although the participating children derived from different backgrounds and neighbourhoods, and are rather representative for the population of Winterthur, our results are not generalizable for suburban or rural schools from other Swiss regions.

## Conclusions

This study used the novel combination of accelerometry and GPS to objectively assess how different activity settings are used by sixth grade pupils from an urban population in Switzerland to engage in PA. Our findings are aligned with previous results and further demonstrate the importance of school and street setting for children to achieve their recommended daily level of MVPA. Therefore, authorities should be aware of the need to provide school settings in which children, and especially girls, are encouraged to be physically active. Safe streets are needed to maintain or enhance levels of active commuting and informal play. Recreational facilities such as parks, playgrounds and sports fields were found to be highly conducive for PA, although infrequently visited. Future interventions should aim to increase the use of these settings.

Further research, conducted within different seasons and including children of different ages from different geographic areas and socio-economic backgrounds, is needed to test the generalizability of the presented results. Future studies should further investigate how socio-economic factors can influence the spatial activity patterns of primary school children. Also more detailed knowledge about the differences between boys and girls is required to reduce the differences between sexes in future interventions. Finally, future work should target on methodological aspects to further improve the accuracy of studies using accelerometers and GPS.
